# Surface Structure Modulation of La_0.6_Sr_0.4_CoO_3_ Films on SrTiO_3_ (001) Substrate under Electrochemical Conditions

**DOI:** 10.1021/acsami.5c11807

**Published:** 2025-10-03

**Authors:** Atsuro Fujisawa, Xuhui Xu, Yuta Ishii, Hidekazu Shimotani, Yuta Inoue, Yuto Miyahara, Kohei Miyazaki, Yusuke Wakabayashi

**Affiliations:** † Department of Physics, Graduate School of Science, 13101 Tohoku University, Sendai 980-8578, Japan; ‡ Center for Basic Research on Materials (CBRM), 52747 National Institute for Materials Science (NIMS), 1-2-1 Sengen, Tsukuba, Ibaraki 305-0047, Japan; § Graduate School of Engineering, 12918 Kyoto University, Nishikyo-ku, Kyoto 615-8510, Japan; ∥ Graduate School of Engineering, Kobe University, 1-1, Rokkodai, Nada, Kobe 657-8501, Japan

**Keywords:** transition metal oxide surfaces, thin films, surface structure, water electrolysis, X-ray diffraction, *in situ* measurements

## Abstract

The surface structure of the La_0.6_Sr_0.4_CoO_3_ film, a typical model water-splitting catalyst, is examined under vacuum and electrochemical conditions using surface X-ray diffraction. The pristine sample has a two-unit-cell-thick strongly polarized SrCoO_3_ layer at the surface, and the surface termination is predominantly a CoO_2_ layer with apical oxygen atoms. After electrochemical treatment, the surface was covered with an additional edge-shared CoO_6_ double layer. The polarization in the SrCoO_3_ region was greatly reduced. These structural changes were accompanied by an increase in the working electrode current, suggesting a strong relationship between surface structure modulation and catalytic activity. Reversible structural modulation induced by the film’s electric potential was observed and was qualitatively understood as atomic displacements caused by the local electric field and change in the Co ionic radii.

## Graphical abstract

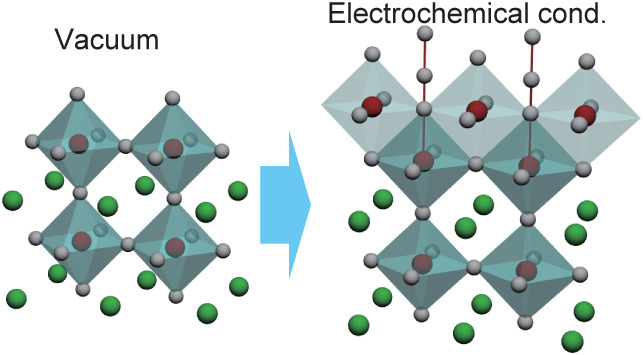

## 1 Introduction

Spontaneous mass transport sometimes plays a significant role in the properties of heterogeneous systems such as at surfaces and grain boundaries. Phase separation and proton conduction are typical examples of mass transport inside a material. In iron passivation, a classical example of chemical reactions at an interface, oxygen vacancies in the iron oxide layer flow toward the surface to make the passive layer thicker.^1–4^ Mass transport in the environment often controls the rate of various chemical reactions at solid–liquid interfaces by limiting the amount of reactant at the interfaces.

In water electrolysis in an alkaline environment, O_2_ is formed from abundant OH^–^, and therefore, the mass transport in the water side is completed immediately. In contrast, mass transport in the electrode side causes the widely observed activation and degradation of electrocatalysts.^5–8^ The surface structure is one of the important factors affecting the oxygen evolution reaction (OER) activity.^9–13^ The importance of the structural information is enhanced for oxides because the oxide structure reflects the electronic states, such as the bonding nature, valency, and orbital and spin states of cations. Oxide catalysts have been extensively studied because of their high environmental stability, including at high temperature, and high degree of freedom to control the chemical and electronic structures.^9,11,13–17^ Spontaneous mass transport causes unintended structural modulation. In addition, the OER at the oxide surface is assumed to involve lattice oxygen in the so-called lattice oxygen-mediated mechanism (LOM)^18^ in which spontaneous mass flow plays an important role. Therefore, *in situ* structural measurements of oxide catalysts are required.

High-resolution surface structure analysis in controlled environments can be achieved using the crystal truncation rod (CTR) scattering method, a surface X-ray diffraction technique.^19,20^ Using this technique, the catalytic reactions have been examined at gas–noble metal^21–23^ and liquid–noble metal interfaces.^24–26^ Regarding oxide catalysts, rutile-type oxides have been studied in detail^12,27–29^ because part of the CTR signal from the rutile structure is solely from oxygen,^27,30^ which makes detailed structural analysis of the oxygen sublattice easier. However, perovskite (001) surfaces do not provide such a signal solely originating from oxygen, which hampers the detailed examination of surface oxygen positions in perovskite oxides under electrochemical conditions.

Recent advances in analytical methods have led to many reports of detailed structures of perovskite surfaces under vacuum conditions.^31–37^ Here, we report the surface structure of La_1–*x*_Sr_*x*_CoO_3_, an active water-splitting catalyst, before and during electrochemical treatment. For this compound, transmission electron microscopy at a vapor pressure of H_2_O of a few Pa, *ex situ* measurements of lattice parameters^8^ and surface structure analysis based on CTR measurements under vacuum without any electrochemical treatment^37^ have been reported. However, an *in situ* surface structure analysis is yet to be reported. We prepared an atomically flat thin film grown on an SrTiO_3_ (001) surface. Under vacuum, the surface was predominantly terminated by CoO_2_ planes with apical oxygen atoms on top of the Co sites. Time evolution of the surface structure was reported in the early stage of the electrochemical process, and once saturated, nearly half of the surface was covered with an additional CoO_2_ layer, i.e., CoO_2_ double-layer termination. The observed CoO_2_ double-layer structure has edge-shared CoO_6_ octahedra, and this favors oxy-hydroxide formation.^38^ Additionally, the electric potential reversibly controls the surface structure, and this process involves surface polarization from oxygen displacement.

## 2 Methods

La_1–*x*_Sr_*x*_CoO_3_ epitaxial films were grown on Nb-doped SrTiO_3_ (001) substrates (10 × 10 × 0.5 mm) using pulsed laser deposition with an Nd:YAG laser. The wavelength, laser power, and repetition rate were 266 nm, 20 mJ, and 2 Hz, respectively. During deposition, the substrate temperature was kept at 600 °C.

The sample was mounted in a vacuum chamber or a sealed electrochemical cell (a schematic view of the cell is shown in Figure S6) filled with 0.1 mol/L KOH aqueous solution. Prior to the cell preparation, the solution was purged with N_2_ gas bubbled for 15 min. The electric potential *V* of the sample was controlled by a potentiostat relative to the Ag/AgCl reference electrode with 3 mol/L KCl aqueous solution. Throughout this paper, all potentials are quoted relative to the Ag/AgCl electrode.

CTR scattering measurements were performed at BL-4C at the Photon Factory, KEK, Japan. A synchrotron X-ray beam was monochromatized by a Si (111) double-crystal monochromator and focused on the sample by a bent cylindrical mirror. A standard four-circle diffractometer was installed on the beamline, and a small two-dimensional pixel array detector (XPAD-S70, imXpad, France) was attached on the 2θ-arm with double-slit optics. The measurements were carefully performed to minimize radiation damage under the electrochemical conditions. All measurements were performed at room temperature.

Quantitative analysis of the CTR intensity profiles was performed using the Bayesian analysis software CTR-structure.^39,40^ In this study, we took the topmost CoO_2_ plane as the origin of the phasing and assumed a Gaussian distribution of the surface height so that the detailed surface structure could be discriminated from the surface roughness (see Section 1 of the Supporting Information for more details on the diffraction theory). The sample structure was characterized under vacuum with a standard procedure reported elsewhere.^35^ Only a subtle modulation of the surface structure was expected to be induced by the applied potential. Therefore, quantitative analysis was performed on the ratio of the intensity modulation caused by the potential; the idea is similar to that for the observation of the electric double layer based on the X-ray reflectivity measurement.^41^

The surface structure model that we constructed is presented in Figure 1. The (001) surface of the perovskite structure can have BO_2_ termination or AO termination. The occupancy parameters of the atoms up to the surface BO_2_ layer (the atoms presented in Figure 1a) are fixed to unity. The topmost Co site among the fully occupied sites is labeled B_top_ as shown in Figure 1. Oxygen sites in the AO plane and BO_2_ plane are called the O_*I*_ and the O_*II*_ sites, respectively. The surface structure models are presented in (b)–(d). On top of the B_top_O_2_ layer, we assumed there was an AO plane (O(1) and A_s_ in (c)), a BO_2_ double-layer structure (O(3) and B_DL_ in (d)), and additional oxygen with reduced occupancy parameters. The surface model structure shown in (d), the BO_2_ double-layer model was constructed based on the reported surface structure of SrTiO_3_;^42^ only half of B_DL_ and O(2), which is the apical oxygen to the B_DL_ site, are occupied to maintain the stoichiometry in the ideal BO_2_ double-layer termination structure (see Section 2 and Figure S1 of the Supporting Information). The distances of O(1)–O(4) and O(1)–O(5) are ∼1.2 Å and ∼2.7 Å, which are the covalent bond length of O–O and the hydrogen bond length of OH–O, respectively. The other transparent spheres in Figure 1c,d show the assumed oxygen atom positions, and their occupancy was less than 10% under any conditions in this study. The O(3) position overlaps A_s_, thus, quantitative discussion on the O(3) site is impossible.

**Figure 1:**
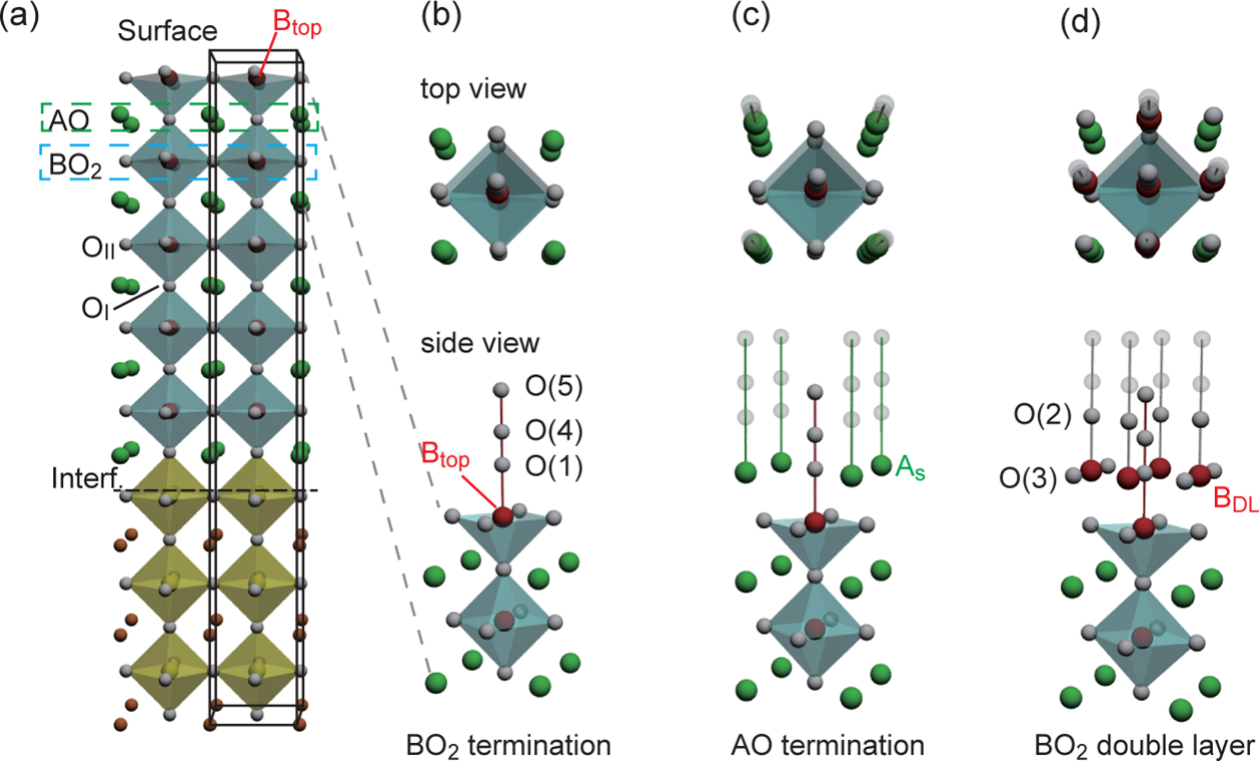
Surface structure model used in this study. Yellow and blue octahedra show TiO_6_ and CoO_6_ octahedra. Green, red, and white spheres in (b)–(d) show the A-site, B-site, and oxygen atoms, respectively. The occupancy parameters of the atoms up to the B_top_O_5_ pyramid are unity. (a) Overall structure of the film. The structural parameters for the atoms within the pillar shown by the black frame are refined: (b) BO_2_ termination, (c) AO termination, and (d) BO_2_ double-layer termination models. Transparent spheres show the assumed oxygen atom positions, and their occupancy was less than 10%.

The measurements were carried out in the order (i) under-vacuum CTR (Figure 2a), (ii) cyclic voltammetry (CV) first run (Figure 3a), (iii) time evolution of CTR (Figure 3b), (iv) potential dependence measurements of CTR at several selected scattering vectors (Figure 3c–f), (v) CV second run (Figure 3a), (vi) *in situ* CTR for surface structure analysis (Figure 2c), and (vii) CV third run (Figure 3a). The time dependence of the potential and working electrode current is presented in Figure S5.

**Figure 2:**
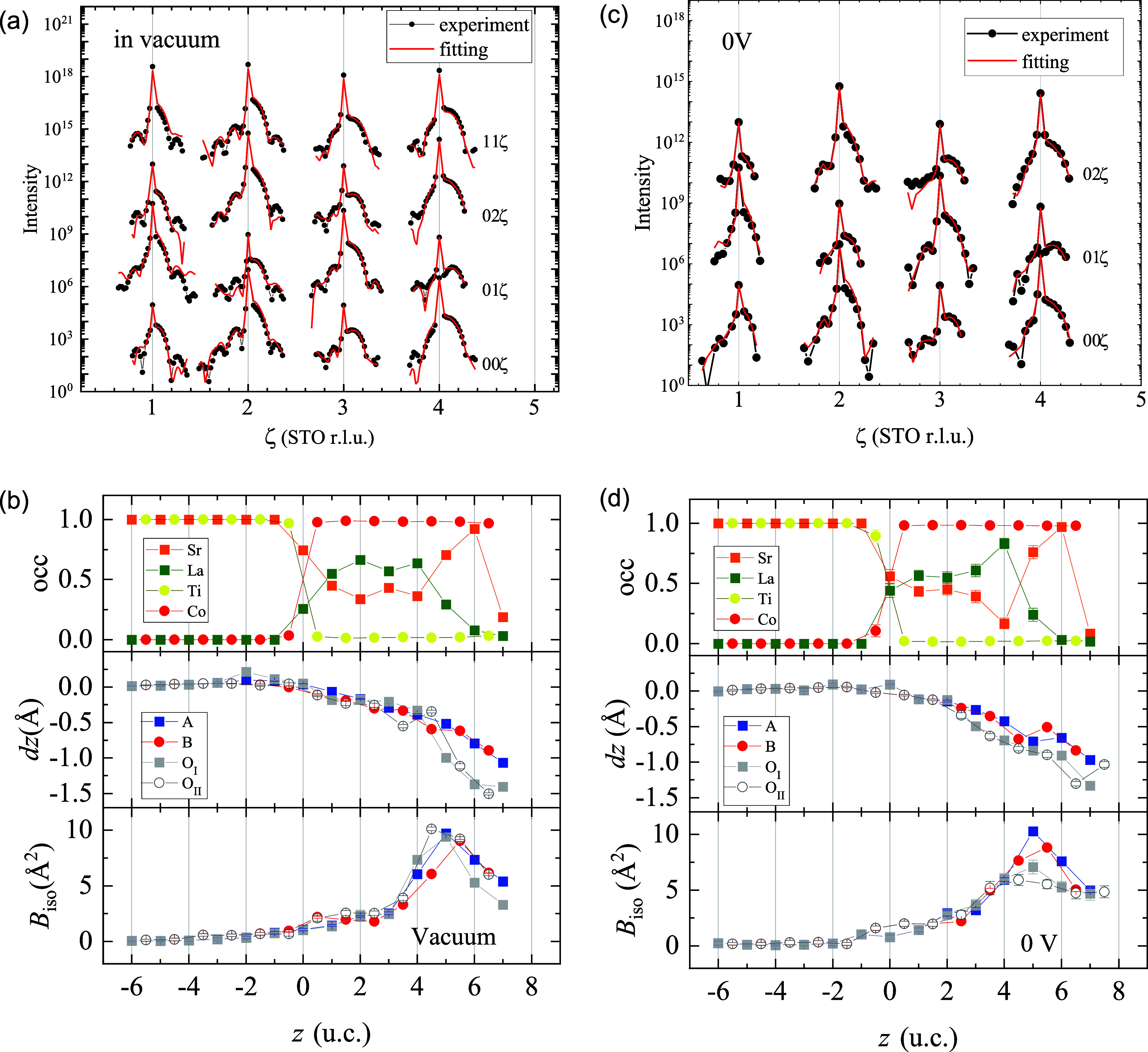
(a) CTR intensity profiles measured under vacuum together with the result of fitting. (b) Depth profile of the obtained structural parameters in the pillar (Figure 1a). *z* = 7 shows the parameters for the A_*s*_ and O(1) sites. (c) CTR intensity profiles in the KOH aqueous solution at 0  V. (d) Obtained structural parameters in the pillar for 0  V data. Structural parameters at the surface (Figure 1b–d) are listed in Table 1.

**Figure 3:**
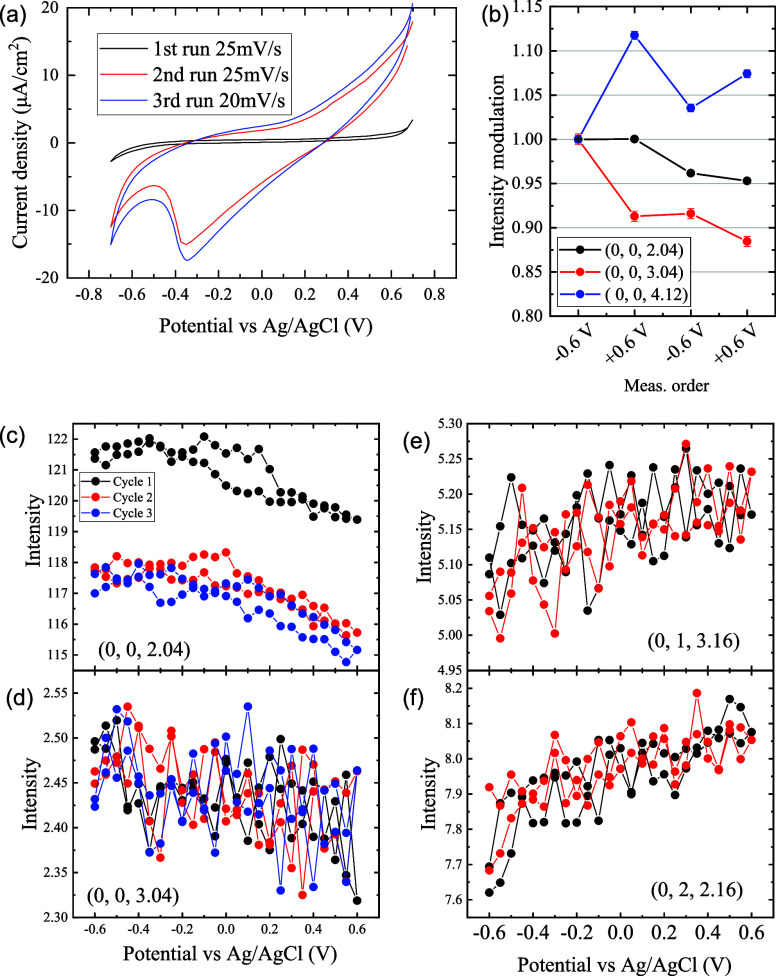
(a) Results of the CV measurements. Initial (1 st run), 1.5 days later (2 nd run), and 2.3 days later (3 rd run). (b) Time evolution of the intensity with switching potential. (c)–(f) XCV profiles measured at (0, 0, 2.04), (0, 0, 3.04), (0, 1, 3.16), and (0, 2, 2.16).

## 3 Results and Analysis

### 3.1 Under-Vacuum Structure

The CTR intensity profiles measured on *hk*ζ-lines under vacuum are presented in Figure 2a together with the results of Bayesian analysis. The obtained structural parameters are presented in Figure 2b. The horizontal axis shows the depth, and the vertical axes show the occupancy, atomic displacement with respect to the substrate lattice *dz*, and isotropic atomic displacement parameter *B*_iso_. The thickness of the film is nearly 7 unit cells, and the Sr concentration for the A-site is 0.4 in the middle of the film, and nearly 1 at the surface. The Sr segregation at the surface was also confirmed by the analysis using the Sr K-absorption edge (see Section 3 of the Supporting Information). In the Sr-concentrated region (*z* ≥ 5), the cations are displaced outward from the oxygen atoms as shown in the *dz* profiles in Figure 2b, meaning that there is electric polarization (or an electric field) at the surface. In the middle of the film, in the La_0.6_Sr_0.4_CoO_3_ region, the relative displacement of the cations and anions disappears, indicating that the inside of the film is metallic. The parameter *B*_iso_ is a measure of the positional fluctuation of each atom from the in-plane lattice-averaged position. In defect-free crystalline samples, *B*_iso_ represents the amplitude of thermal vibrations and its typical value at room temperature is 0.5 Å^2^. In thin-film specimens, it often primarily reflects disorder arising from lattice defects and atomic intermixing and can reach relatively large values, on the order of 5 Å^2^.^35,36^ The *B*_iso_ parameters greatly increase around *z* = 5, which coincides with the interface between La_0.6_Sr_0.4_CoO_3_ and SrCoO_3_ formed by the Sr segregation.

Other structural parameters for the atoms close to the surface are given in Table 1. The A_s_ and B_DL_ occupancies were 22(2)% and 3(1)%, respectively, which means that 72(4)% of the as-grown surface is BO_2_ termination (Figure 1b). The O(1) occupancy is 99(1)%, meaning that the Co at the surface is octahedrally coordinated.

**Table 1:** Selected Surface Structure Parameters for Vacuum and 0  V Conditions^a^

Parameter	vacuum	0 V	–0.6 V	+0.6 V
occ O(1)	0.99(1)	0.93(5)	–0.02(2)	+0.03(2)
occ O(2)	–	0.19(4)	–0.04(3)	+0.07(3)
occ O(4)	–	0.65(8)	–0.10(8)	+0.04(6)
occ O(5)	–	0.24(7)	+0.08(8)	–0.02(6)
occ B_DL_	0.03(1)	0.22(2)	+0.00(1)	+0.00(1)
occ A_s_	0.22(2)	0.11(3)	+0.00(1)	+0.00(1)
B_top_–B_DL_ (Å)	–	1.87(4)	–0.02(4)	–0.13(2)
B_top_–O(1) (Å)	1.44(4)	1.46(4)	+0.02(4)	+0.08(6)
B_top_–O_*I*_ (Å)	2.43(5)	2.02(5)	–0.09(6)	+0.13(4)
B_DL_–O(2) (Å)	–	1.84(7)	–0.04(14)	+0.02(6)
B_DL_–O_*II*_ (Å)	–	2.34(6)	–0.13(6)	–0.02(5)
O(1)–O(4) (Å)	–	1.04(5)	+0.09(5)	–0.05(7)
O(1)–O(5) (Å)	–	2.80(6)	+0.1(2)	–0.16(18)

a Those for −0.6 V and +0.6 V conditions are expressed by the relative value with respect to the 0 V condition. O(2), O(3), and O(5) were not taken into account in the analysis for the under-vacuum condition. B_top_–B_DL_ is the height of the B_DL_ site measured from the B_top_ site.

### 3.2 Potential Dependence of the Surface Structure

The CV results are listed in Figure 3a. The potential range of the CV measurement was carefully selected to avoid bubble formation caused by the large currents. If bubbles form on the electrode surface due to water electrolysis, they remain on the surface because the cell is sealed. This alters the amount of water in the X-ray path, leading to changes in absorption and a significant increase in signal intensity. These variations would not only prevent reliable data analysis but also make it difficult to maintain liquid contact with the sample surface. Note that the vertical axis in the voltammogram spans a very narrow range. For the pristine sample, the voltammogram shows few features except for an increase in the current at +0.7 V, on the OER side, and −0.7 V, on the oxygen reduction reaction (ORR) side. Based on this profile, we examined the surface structure at 0 V and ± 0.6 V. After 1.5 days of X-ray measurements, CV was recorded again, and the current was greatly increased. A similar change in CV caused by electrochemical operation was also reported in ref. 8; they attributed the increase in current as a formation of an active electrode surface under OER conditions. Quantitatively, it was reported that the working electrode current is suppressed in ultrathin films.^43^ The overall feature of the CV remains unchanged until the end of the experiment (2.3 days from the first CV measurement, third run). The time evolution of the working electrode current during the experiment is presented in Figure S5. There was no detectable change in the working electrode current caused by X-ray irradiation in these measurements, indicating that the photochemical reaction is negligible

Figure 3b shows the time evolution of the CTR intensity measured at (0, 0, 2.04), (0, 0, 3.04), and (0, 0, 4.12). The measurement was done in the order of −0.6, + 0.6, −0.6, and +0.6 V. The intensity depends on both the time and potential, and the dependency varies as a function of the scattering vector. The potential dependence is further examined by measuring the intensity as a function of potential at a fixed scattering vector; we call such measurements as XCV measurements. XCV profiles measured at (0, 0, 2.04), (0, 0, 3.04), (0, 1, 3.16), and (0, 2, 2.16) are presented in Figure 3c–f. Reproducible potential dependence of the intensity and, therefore, the surface structure was observed. Only the first XCV cycle at (0, 0, 2.04) differs from the second and third cycles, showing that the time evolution of the surface structure stops during this XCV measurement.

We performed surface structure analysis on the 0 V data measured after the XCV measurements. The second run of the CV measurement shown in Figure 3a was conducted just before the CTR measurement at 0 V. The results are presented in Figure 2c,d and Table 1. The overall feature of the film structure shown in Figure 2d is similar to that of the under-vacuum structure (Figure 2b). There is no observable change in the *c*-lattice spacing within the film region. This stands in contrast to previous *ex situ* lattice spacing measurements of thick films before and after electrochemical treatment,^8^ which reported clear lattice expansion. In that study, long-term operation was carried out until the end of the electrode’s lifetime, and lattice parameter changes were observed in the deactivated sample. The slight change in lattice spacing in the present case suggests that the damage responsible for catalytic deactivation is minor. The *z* positions of oxygen in the Sr-concentrated region are close to those of the cations at 0 V, meaning that the surface polarization observed under vacuum is reduced. This structural change is shown in Figure 2b,d and in Table 1 (B_top_–O_*I*_ of the Supporting Information). The occupancy of A_s_ and B_DL_ was 11(3)% and 22(2)%, respectively. This result means that BO_2_ double-layer termination, which was effectively not found in the under-vacuum measurement, covers 44% of the surface (note that the maximum occupancy parameters of B_DL_ and O(2) sites in the BO_2_ double-layer surface are 0.5, see Section 2). The occupancy of O(2) was comparable to that of B_DL_, meaning that most B_DL_ sites are octahedrally coordinated.

The observed intensity modulation induced by the applied potential is typically smaller than 5% (see Figure S4) and therefore, when we plot the intensity measured at ± 0.6 V on the log scale, the results completely overlap with the 0 V result shown in Figure 2c. It should be noted that the typical uncertainty of the CTR intensity distribution is 20%,^39^ which is mainly caused by optical misalignment. This uncertainty is apparent when comparing the intensity between two distant *Q* points. The uncertainty for the intensity measured at the same *Q* point is much smaller, as shown in the XCV measurements. To derive the structural modulation caused by the applied potential, we calculated corrected intensity $I^{\prime V}\left(Q\right)$ :

1 $$I^{\prime V}\left(Q\right)=\frac{I_{\mathrm{calc}}^{0V}\left(Q\right)}{I_{\exp}^{0V}\left(Q\right)}I_{\exp}^{V}\left(Q\right)$$

where $I_{\exp}^{V}\left(Q\right)$ denotes the measured intensity at potential *V*, and $I_{\mathrm{calc}}^{0V}\left(Q\right)$ denotes the calculated intensity based on the obtained 0 V structure. The prefactor $I_{\mathrm{calc}}^{0V}\left(Q\right)/I_{\exp}^{0V}\left(Q\right)$ corrects the Q -dependent error caused by the optical misalignment; $I^{\prime V}\left(Q\right)$ is free from the uncertainty caused by optical misalignment, which allows us to examine the effect of the potential on the film structure. The results of the Bayesian analysis performed for $I^{\prime V}$ are nearly the same as those for the 0 V structure except for the top surface region. The relative change in the surface structure parameters from the 0 V structure is given in Table 1. Because the potential dependence of the CTR intensity is very small, the observed surface structural modulation induced by the potential is also minute.

The potential dependence of the surface structure is schematically presented in Figure 4. The oxygen displaces inward as the potential increases. In addition, B_DL_ ions also displace inward and the B_DL_–O­(2) distance is unchanged. The O(2) occupancy increases. The structural parameters for the atoms inside are nearly independent of the potential.

**Figure 4:**
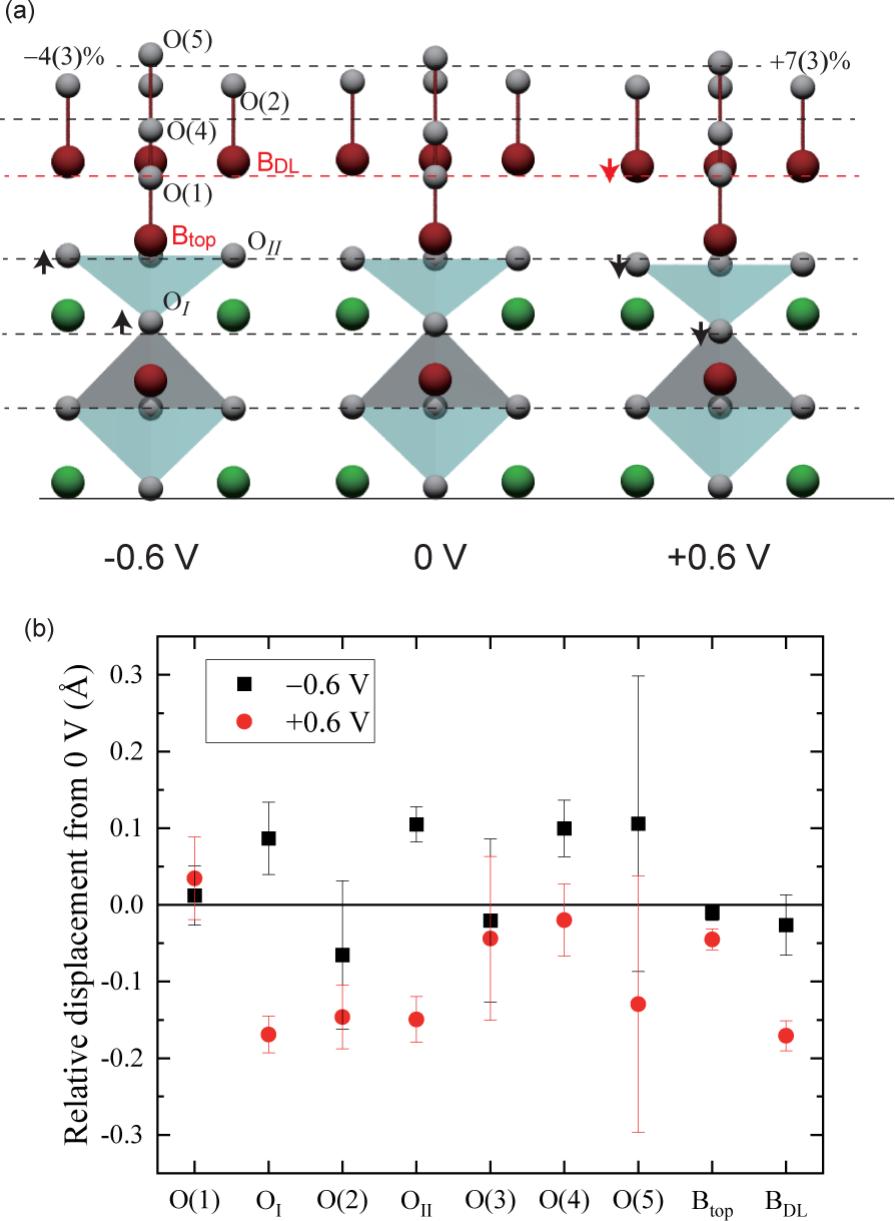
(a) Schematic view of the potential (*V* vs Ag/AgCl electrode) dependence of the surface structure. Horizontal dashed lines are to guide the eye. (b) Relative atomic displacement with respect to the 0 V structure.

## 4 Discussion

First, the reliability of the analyzed structure is examined. The uncertainty of all structural parameters reported in this paper was estimated from the probability density distributions obtained via Bayesian inference based on the experimentally measured intensity profiles. As shown in Figure 2a, the CTR intensity profiles measured in vacuum span nearly the entire range along the rod direction. Although the signal near ζ=n+0.5 , where *n* is an integer, is generally too weak to detect, the intensity around (00ζ) with ζ≃1.5 is observed almost continuously. Consequently, depth-sensitive structural information was obtained for all wavevectors within the Brillouin zone, allowing the Bayesian analysis to yield probability distributions for each structural parameter. In contrast, the CTR profiles measured at 0 V (Figure 2c) exhibit wide gaps near ζ=n+0.5 , resulting from X-ray absorption and increased background from the solution. These gaps lead to a loss of information associated with 2-fold periodic structures. Although this affects the reliability of the depth profile of the lattice spacings, the average spacing over two or more unit cells remains robust. The surface termination structure model is well localized in real space, and its corresponding information in reciprocal space is broadly distributed across a wide ζ range. Therefore, the derived surface termination structure is considered reliable. Potential-dependent structural changes were analyzed based on subtle intensity variations shown in Figure S4, where the gaps are wider than those in Figure 2c. These wide gaps result in the large parameter uncertainties shown in Figure 4b.

Next, we compare our result with the previously reported La_1–*x*_Sr_*x*_CoO_3_ film structure^37^ (*x* = 0.2, under-vacuum measurement without any electrochemical treatment). Ref. 37 reported Sr segregation at the surface, the formation of a LaCoO_3_ layer underneath the dense Sr layer, SrCoO_3_ particles on the surface, and surface polarization. The Sr segregation at the surface is observed in our work, meaning that Sr segregation is quite common in La_1–*x*_Sr_*x*_CoO_3_ film growth in the pulsed laser deposition process. Similar Sr segregation was also reported for La_1–*x*_Sr_*x*_MnO_3_ film.^44^ The orientation of the surface polarization reported in ref. 37 is opposite to what we observed. We found short B–O_*I*_ on the surface side as shown in Figure 2b, while they reported this on the bulk side.

Local polarization at the oxide interface is controlled by the spatial distribution of the chemical species. For example, the local electric field at the interface between LaMnO_3_ and LaNiO_3_ points to LaNiO_3_.^45^ In the present case, our sample and that reported in ref. 37 have different spatial distribution of Sr. Our sample does not have a LaCoO_3_ layer underneath or SrCoO_3_ particles on top, which cause different local electric fields. There is no apparent polarization inside our film, which suggests metallic behavior in the middle of the film electrode.

In this study, a BO_2_ double-layer structure (Figure 1d) was developed after 1.5 days of electrochemical treatment. B_DL_O_6_ octahedra are edge-shared with neighboring B_top_O_6_ octahedra.

Edge-shared structures have also been suggested in the electrochemically formed amorphous layer grown on the surface of the highly active catalyst Ba_0.5_Sr_0.5_Co_1–*x*_ ${\mathrm{Fe}}_{x}O_{3-\delta }$ .^5,6^ Thus, the catalytic activity of La_1–*x*_Sr_*x*_CoO_3_ may also be enhanced by the formation of this structure. The formation of the edge-shared BO_6_ octahedra structure affects the electronic energy levels and the degree of steric hindrance, which alters the catalytic activity.^46^ With a support of noble metals, it favors oxy-hydroxide formation,^38^ which helps OER at the surface. In the present case, the CV results show a considerable increase in the electric current (Figure 3a) after developing the BO_2_ double layer at the surface. A similar change in CV was also reported in ref.  ^8^, in which redox activity was increased by forming an active surface under OER conditions. They attributed this increase in activity to the formation of CoO­(OH) primarily based on the observation of two kinds of Co and O in the X-ray photoelectron spectra. Our observation exhibits the coexistence of edge-shared and corner-shared CoO_6_ octahedra, which involve two kinds of Co and O sites. It should also be noted that our results do not exclude the formation of Co–OH bonds, as X-ray diffraction is insensitive to hydrogen atoms.

The potential dependence of the atomic displacement presented in Figure 4 is moderate. Increased potential causes inward atomic displacement. This tendency is in accordance with a simple view that a higher potential makes the electric field point outward, which results in the inward displacement of oxygen. The volume of B_top_O_6_ octahedra is increased by 8(±6)% when the potential is increased from −0.6 to +0.6 V. This volumetric change suggests that the Co ion valency is reduced^47,48^ or a higher spin state is stabilized^47^ with applied potential. We expect a similar potential dependence for the B_DL_ site, but the large uncertainty in the obtained structural parameter does not allow us to confirm this.

Based on the CV results in Figure 3a, the BO_2_ double-layer termination surface has a larger double-layer capacity and an ORR peak at −0.3 V. The peak was attributed to oxygen intercalation.^18^ Although oxygen intercalation may be observed through the structure, there was no detectable change in structure around −0.3 V; this is because of the tiny total amount of charge. The increase in capacity is usually attributed to an increase in surface area. However, this change in double-layer capacity is not caused by the increase in the surface area because the CTR results show little change in the surface roughness. The increase in the capacity corresponds to the space charge of ∼2 electrons per unit cell area around the interface. Some of this space charge is explained by the change in the B_top_ site valency and the O(2) occupancy discussed above. In addition, there is an expected change in the B_DL_ site valency and proton addition/removal should contribute, but this is not directly observed in our X-ray investigation. The voltammogram shows a positive slope after electrochemical treatments, which implies that the conductivity of the film increased. This change in conductivity was reflected in the structure, as evidenced by reduced polarization in the very surface region (5≤ *z* ≤ 7 in Figure 2b,d).

Enhanced *B*_iso_ parameters around the surface (Figure 2b,d) can result from a random electric field caused by the local arrangement of La/Sr or vacancies. However, a large *B*_iso_ parameter sometimes implies a reduced occupancy through the parameter coupling. If so, the enhanced *B*_iso_ of O_*I*_ and O_*II*_ seen in Figure 2b suggests a large number of oxygen vacancies around the surface, which are filled during the electrochemical treatment. Such a migration of oxygen vacancies at the surface under electrochemical conditions suggests that the reaction process involves lattice oxygen at the surface, in accordance with the LOM. The reduction of surface polarization and oxygen *B*_iso_ around the surface suggests active atomic flow around the surface in a 1 nm range induced by the electrochemical conditions. This finding gives an idea of the range and magnitude of the mass flow around the surface under electrochemical conditions.

## 5 Conclusion

The surface structure of the CoO_2_-terminated La_1–*x*_Sr_*x*_CoO_3_ film grown on SrTiO_3_ was examined under vacuum and electrochemical conditions. Sr segregation was found at the first two AO planes, forming an ultrathin SrCoO_3_ layer at the surface. This Sr segregation was stable under electrochemical treatment. The surface structure was modulated during the electrochemical treatment to form BO_2_ double-layer termination, which involves edge-shared CoO_6_ octahedra. Strong electric polarization was observed in the pristine sample and it was reduced under electrochemical conditions. The isotropic atomic displacement parameters *B*_iso_ of the atoms in the range of 1 nm from the surface are considerably larger than those in the interior. This tendency is unchanged for cations under electrochemical treatment. For oxygen, in contrast, the increase in *B*_iso_ at the surface is reduced after the formation of the BO_2_ double-layer surface. The reduction of surface polarization and oxygen *B*_iso_ around the surface suggests active atomic flow in the range around 1 nm from the surface, induced by the electrochemical conditions.

## Supplementary Material



## Notes

Support_Fujisawa.pdf: Descriptions of (Sec. 1) diffraction theory, (Sec. 2) surface model for surface structure analysis, (Sec. 3) examination of Sr segregation, (Sec. 4) results of fitting for deriving potential dependence of the surface structure, (Sec. 5) time dependence of the working electrode current during the synchrotron experiment, and (Sec. 6) schematic of the sample cell (PDF)
