# Supplementary material for: Surface Structure Modulation of La0.6Sr0.4CoO3 Films on SrTiO3 (001) Substrate under Electrochemical Conditions
Source: ACS Appl Mater Interfaces. 2025 Oct 3;17(41):57603–10. doi: 10.1021/acsami.5c11807 (PMC12532094; doi:10.1021/acsami.5c11807)
Supplement: Supplementary file 1 [file am5c11807_si_001.pdf]

Supporting Information
Surface Structure Modulation of $La_{0.6}Sr_{0.4}CoO_3$ Films on $SrTiO_3$ (001) Substrate under Electrochemical Conditions

Atsuro Fujisawa,[†] Xuhui Xu,[†] Yuta Ishii,[‡] Hidekazu Shimotani,[†] Yuta Inoue,[¶] Yuto Miyahara,[¶] Kohei Miyazaki,[¶,§] and Yusuke Wakabayashi[*,†]

†*Department of Physics, Graduate School of Science, Tohoku University, Sendai 980-8578, Japan*

‡*Center for Basic Research on Materials (CBRM), National Institute for Materials Science (NIMS), 1-2-1 Sengen, Tsukuba, Ibaraki 305-0003, Japan*

¶*Graduate School of Engineering, Kyoto University, Nishikyo-ku, Kyoto 615-8510, Japan*

§*Graduate School of Engineering, Kobe University, 1-1, Rokkodai, Nada, Kobe, 657-8501, Japan*

E-mail: wakabayashi@tohoku.ac.jp

1 Surface roughness

In general, the X-ray scattering amplitude at scattering vector $\boldsymbol{Q}$, $F(\boldsymbol{Q})$ is given by

$$F(\boldsymbol{Q}) = \sum_j f_j \exp(i\boldsymbol{Q} \cdot \boldsymbol{R}_j),$$

where f_j and R_j are the scattering factor and position of the j-th atom. When considering a surface, the sample can be regarded as a bundle of the pillar presented in Fig. 1 in the main text. The pillars are well ordered in-plane, and have some positional distribution out-of-plane ('height'). The scattering amplitude can be written using the scattering amplitude from a pillar, $F_{\text{pil}}(\boldsymbol{Q})$,

$$F_{\text{pil}}(\boldsymbol{Q}) = \sum_j^{\text{pillar}} f_j \exp(i\boldsymbol{Q} \cdot \boldsymbol{R}_j) \tag{1}$$

$$F(\boldsymbol{Q}) = \sum_{n_x, n_y} F_{\text{pil}}(\boldsymbol{Q}) \exp\{2\pi i[h \cdot n_x + k \cdot n_y + l \cdot Z(n_x, n_y)]\}, \tag{2}$$

where $Z(n_x, n_y)$ is the surface height at the in-plane position (n_x, n_y). Since h and k are integers, $\exp[2\pi i(h \cdot n_x + k \cdot n_y)]$ is one. Now we assume that the surface height exhibits a Gaussian distribution. Then the amplitude is

$$F(\boldsymbol{Q}) = \sum_{n_x, n_y} F_{\text{pil}}(\boldsymbol{Q}) \exp[2\pi i l \cdot Z(n_x, n_y)] \tag{3}$$

$$= N \sum_Z P(Z) F_{\text{pil}}(\boldsymbol{Q}) \exp[2\pi i l \cdot Z], \tag{4}$$

where N is the number of in-plane unit cells, $P(Z) \propto \exp[-Z^2/\sigma^2]$ is the probability of the surface being height Z, and σ is the measure of surface roughness. Using this form of the scattering amplitude, the surface structure is examined by analyzing the structural parameters constructing $F_{\text{pil}}(\boldsymbol{Q})$. Our Bayesian analysis showed that σ was nearly constant under all experimental conditions in the present study.

2 BO_2 double layer model

Fig. S1 shows a schematic view of the BO_2 double layer model. (a) shows the atoms used in our analysis, and is essentially the same as Fig. 1(d) of the main text. This model retains the four-fold symmetry of the $SrTiO_3$ substrate surface. (b) and (c) show the actual surface structure of the BO_2 double layer termination. The four-fold symmetry is broken. Only half of the $B_{DL}O_6$ octahedra in (a) are occupied. The structures in (b) and (c) are related by 90° rotation about the c-axis through the substrate B-site.

The B_{DL}–O plane shown in (a) contains two B atoms and two O atoms in an area of (3.905 Å)^2, and this is denser than the bulk BO_2 layer. The structure in (b) and (c) contains one B atom and two O atoms, which is the same as the bulk BO_2 layer. Therefore, the upper limit of the occupancy of the B_{DL} site (and O(2) site) is 0.5, while that of the O(3) site is 1. As presented in (b) and (c), $B_{DL}O_6$ octahedra are edge-shared with $B_{top}O_6$ octahedra.

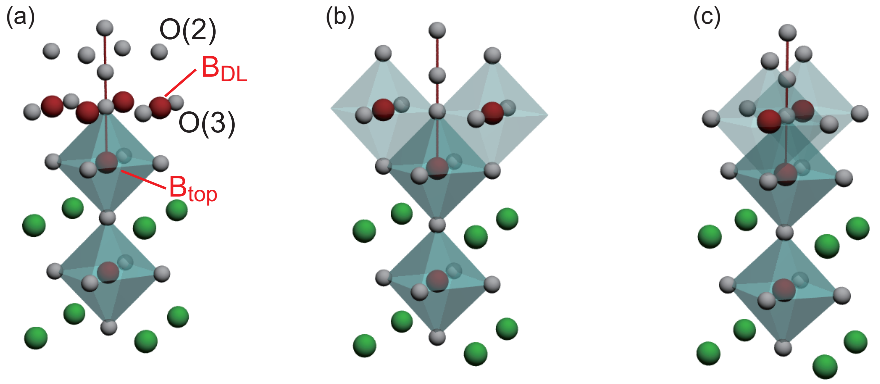

Figure S1: (a) BO_2 double layer model. (b) and (c) Actual surface structure of BO_2 double layer termination.

3 Sr segregation examined by anomalous scattering

To examine the Sr concentration distribution, we measured CTR scattering at 16.05 keV and 16.08 keV, close to the Sr K absorption edge energy (16.095 keV), and 15.8 keV, off resonant condition. The intensity distribution is presented in Fig. S2(a). The intensity

modulation $(I_E - I_{E_0})/(I_E + I_{E_0})$, where I_E is the intensity measured at X-ray energy E and $E_0 = 15.8$ keV, is presented in Fig. S2(b). The anomalous scattering factor changes gradually below the absorption edge, therefore, the intensity modulation for 16.05 keV (black) and 16.08 keV (red) should be similar, and the red plots should be slightly larger than the black plots in most of the profile. The result clearly shows these features, meaning that the intensity modulation of a few % can be discussed based on our experimental result.

Using these data, we analyzed the film structure based on the procedure in Ref. 1. The intensity profiles measured around the absorption edge provide additional information on the elemental distribution. The result is presented in Fig. S3. Sr segregation in the sample is also observed, and the overall features are similar to those in Fig. 2(b) in the main text.

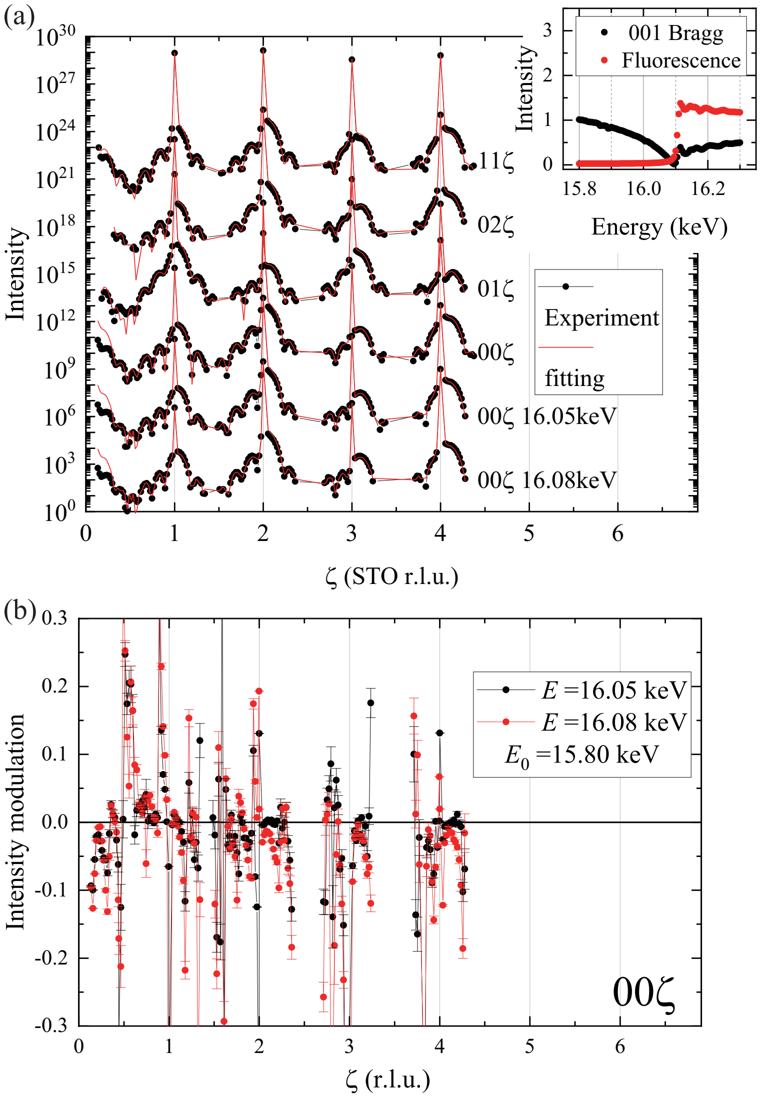

Figure S2: (a) CTR intensity profiles measured under vacuum and the result of fitting. The energy of the incident X-rays was 15.8 keV unless otherwise noted. (inset) Energy spectra of $SrTiO_3$ 001 Bragg reflection (black) and fluorescence (red). (b) Intensity modulation on the 00ζ rod. $E_0 = 15.8$ keV and $E = 16.05$ keV (black) and 16.08 keV (red).

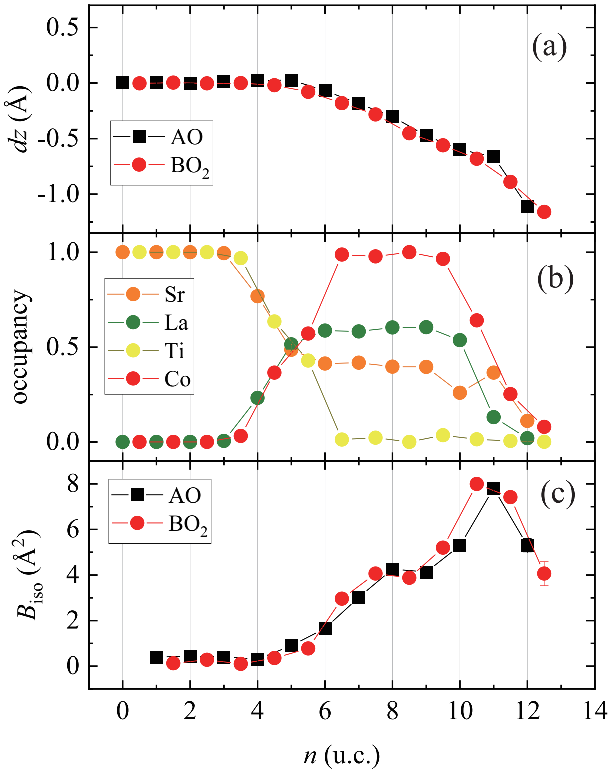

Figure S3: Obtained structural parameters as a function of depth. (a) Atomic displacement with respect to the substrate lattice, dz, (b) occupancy, and (c) isotropic B parameter.

4 Intensity modulation as a function of potential

The intensity modulation $(I^V - I^{0\ \mathrm{V}})/(I^V + I^{0\ \mathrm{V}})$ on (00ζ), (01ζ), and (02ζ) for $V = +0.6$ V and -0.6 V is presented in Fig. S4. The magnitude of the modulation is typically 5 % or less, and the calculation reproduces the main features of the modulation caused by potential control.

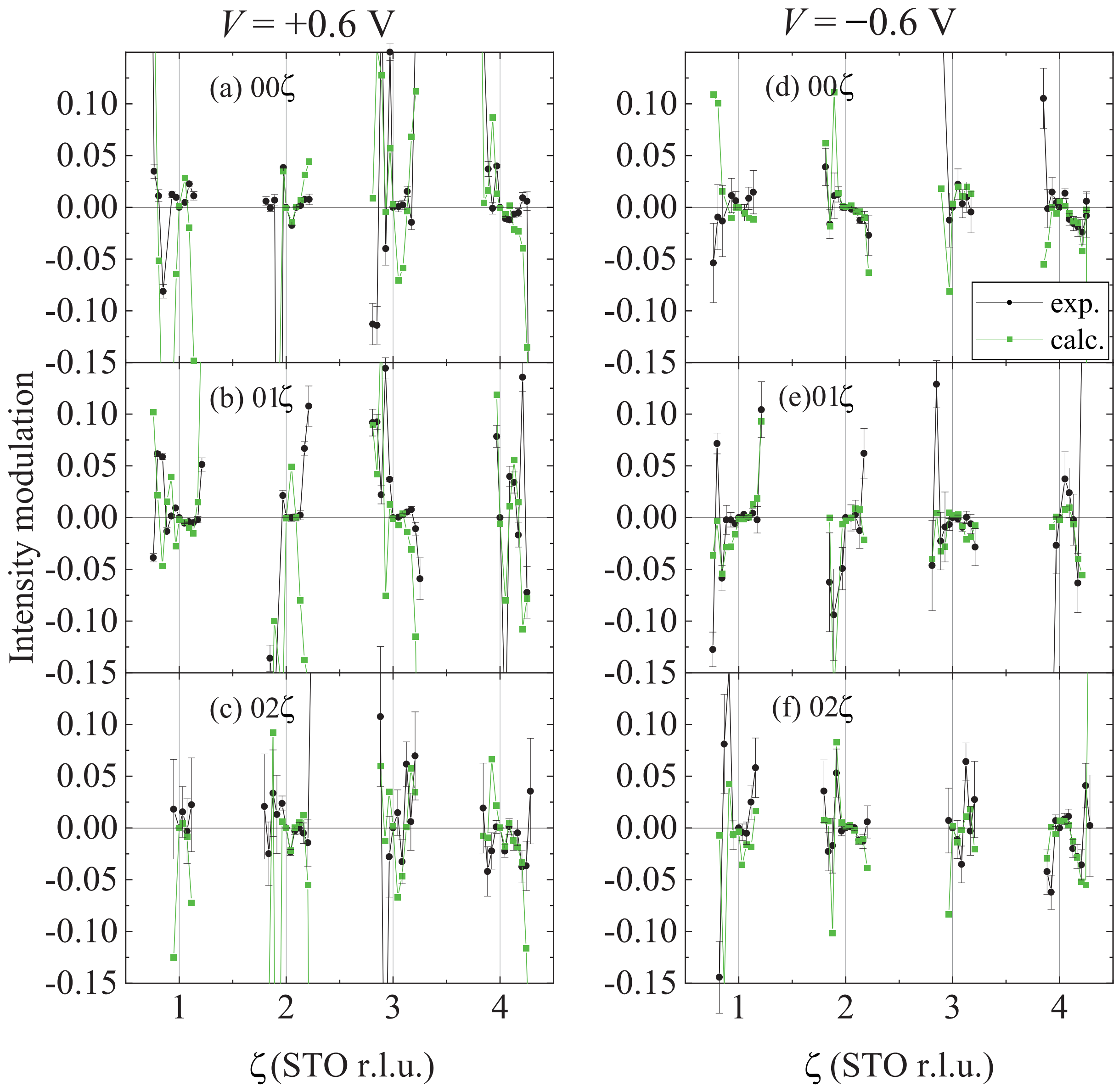

Figure S4: Intensity modulation (a) 00ζ, (b) 01ζ, and (c) 02ζ for $V = +0.6$ V and (d)–(f) $V = -0.6$ V. Calculated values are also plotted.

5 Time dependence of the working electrode current

The sample potential was controlled during the X-ray experiments. The time dependence of the potential and working electrode current is presented in Fig. S5. For the CTR measurements, the potential was switched between $+0.6$ V and -0.6 V for each scattering vector (Fig. 3(b)) or for each rod (Fig. 2(c)).

Fig. S5 shows the timeline of the experiment. During the CTR measurements, we employed an attenuator only when measuring near the substrate's Bragg reflections to reduce the incident X-ray intensity by a factor ranging from 10^2 to 10^7. If any photoelectrochemical reaction had occurred, it would have manifested as a change in current. As illustrated in Fig. S5, there is no detectable current change caused by the X-ray beam. The surface structure gradually changes until the beginning of the XCV measurements. The under-vacuum experiment was performed prior to the electrochemical experiment.

6 Electrochemical cell for diffraction experiments

A schematic view of the electrochemical cell used for the diffraction experiments is shown in Fig. S6. The cell contained a Pt electrode and Ag/AgCl reference electrode. It was filled with 0.1 mol/L KOH aqueous solution to cover the working electrode surface with a thin ($\sim$ 0.1 mm) layer. The liquid was sealed by a polyimide film and the X-ray beam passes through the thin liquid layer.

References

(1) Anada, M.; Sakaguchi, S.; Nagai, K.; Kitamura, M.; Horiba, K.; Kumigashira, H.; Wakabayashi, Y. Local polarization and valence distribution in $LaNiO_3/LaMnO_3$ heterostructures. *Phys. Rev. B* **2021**, *104*, 085111.

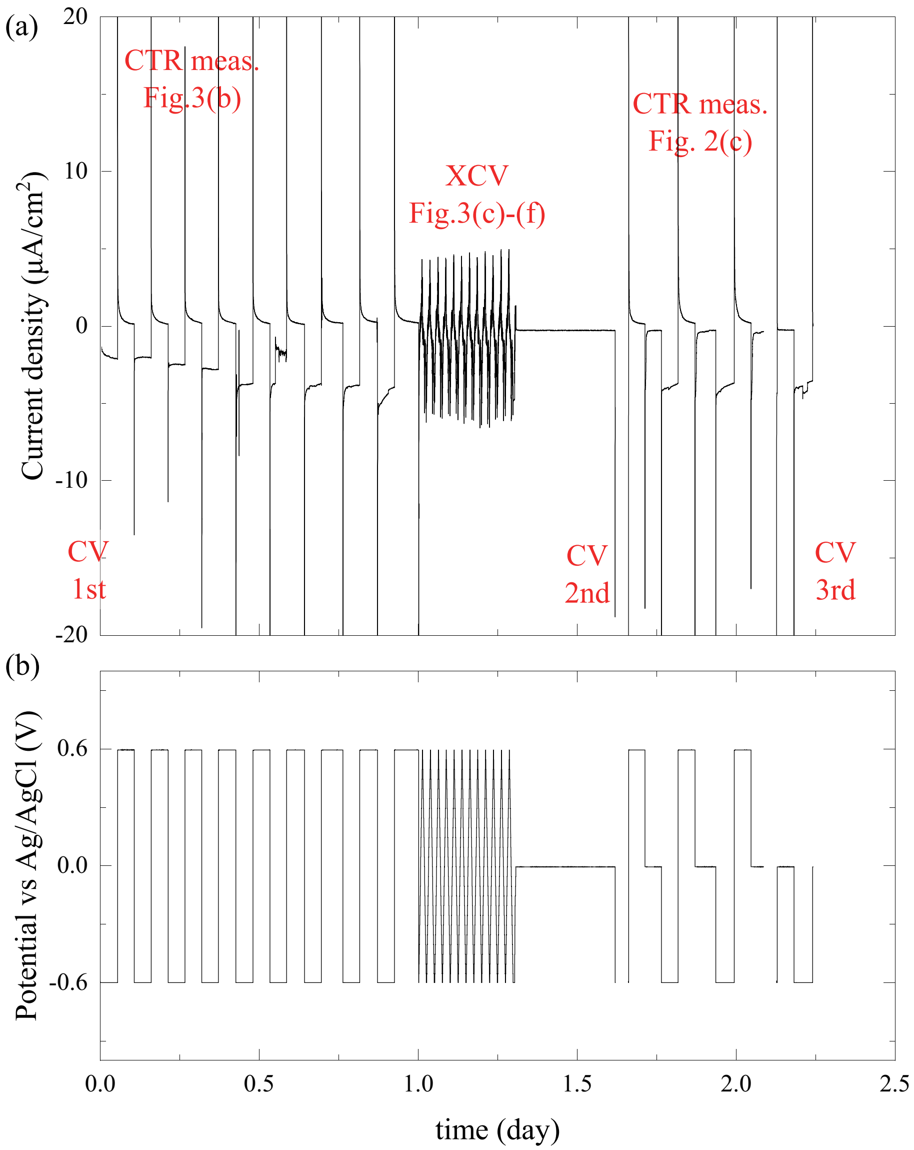

Figure S5: (a) Time evolution of the working electrode current and (b) potential.

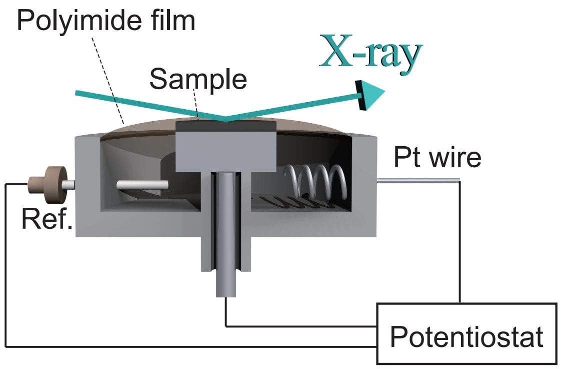

Figure S6: Schematic view of the electrochemical cell used for the diffraction experiments.
